# The Effectiveness of Various Types of Electrical Stimulation of the Spinal Cord for Chronic Pain in Patients with Postherpetic Neuralgia: A Literature Review

**DOI:** 10.1155/2023/6015680

**Published:** 2023-03-24

**Authors:** Emil Isagulyan, Vasily Tkachenko, Denis Semenov, Svetlana Asriyants, Evgeny Dorokhov, Elizaveta Makashova, Karina Aslakhanova, Alexei Tomskiy

**Affiliations:** ^1^Burdenko Institute of Neurosurgery, National Medical Research Center for Neurosurgery Named after Academician N. N. Burdenko, 4th Tverskaya-Yamskaya Street 16, Moscow 125047, Russia; ^2^Central State Medical Academy of Russian Federation, Marshalla Timoshenko Street, 19, Moscow 121359, Russia

## Abstract

**Introduction:**

Postherpetic neuralgia (PHN) is a severe condition that remains a challenge to treat. Spinal cord stimulation (SCS) is used in cases of insufficient efficacy of conservative treatment. However, in contrast to many other neuropathic pain syndromes, there is a huge problem in reaching long-term stable pain relief in patients with PHN using conventional tonic SCS. The objective of this article was to present a review of the current management strategies of PHN, their efficacy, and safety.

**Materials and Methods:**

We searched for articles containing the keywords “spinal cord stimulation AND postherpetic neuralgia,” “high-frequency stimulation AND postherpetic neuralgia,” “burst stimulation AND postherpetic neuralgia” and “dorsal root ganglion stimulation AND postherpetic neuralgia” in Pubmed, Web of Science, and Scopus databases. The search was limited to human studies published in the English language. There were no publication period limitations. Bibliographies and references of selected publications on neurostimulation for PHN were further manually screened. The full text of each article was studied once the abstract was analyzed by the searching reviewer and found appropriate. The initial search yielded 115 articles. Initial screening based on abstract and title allowed us to exclude 29 articles (letters, editorials, and conference abstracts). The full-text analysis allowed us to exclude another 74 articles (fundamental research articles, research utilizing animal subjects, and systemic and nonsystemic reviews) and results of PHN treatment presented with other conditions, leaving 12 articles for the final bibliography.

**Results:**

12 articles reporting on the treatment of 134 patients with PHN were analyzed, with a disproportionally large amount of traditional SCS treatment than that to alternative SCS: DRGS (13 patients), burst SCS (1 patient), and high-frequency SCS (2 patients). Long-term pain relief was achieved in 91 patients (67.9%). The mean VAS score improvement was 61.4% with a mean follow-up time of 12.85 months. Although the number of patients in alternative SCS studies was very limited, almost all of them showed good responses to therapy with more than 50% VAS improvement and reduction of analgesic dosage. The article contains a review analysis of 12 articles concerning the current methods of treatment for postherpetic neuralgia including conservative treatment, spinal cord stimulation, and novel neuromodulation strategies. Available information on the pathophysiology of PHN and the effect or stimulation on its course, together with a number of technical nuances concerning various types of neurostimulation are also elucidated in this article. A number of alternative invasive treatments of PHN are also discussed.

**Conclusions:**

Spinal cord stimulation is an established treatment option for patients with pharmacologically resistant PHN. High-frequency stimulation, burst stimulation, and dorsal root ganglion stimulation are promising options in the management of PHN due to the absence of paresthesias which can be painful for patients with PHN. But more research is still required to recommend the widespread use of these new methods.

## 1. Introduction

Herpes zoster is a viral infection caused by human alphaherpesvirus 3, also known as varicella-zoster virus (VZV), the same virus that causes chickenpox in children. After the acute phase of inflammation, the virus reaches the sensory nervous system and remains latent in trigeminal or dorsal root ganglions for a long period of time. Reactivation of the VZV happens with advancing age or immunosuppression and leads to the development of acute herpes zoster. Pain in affected regions can remain even after the rash resolves. This condition is known as postherpetic neuralgia (PHN) [1]. PHN is a condition that significantly lowers the quality of life and is difficult to treat with medications [2]. Herpes zoster is a relatively common disease; the estimated incidence of acute herpes infection in the European population varies from 1.2 to 5.2 per 1000 people per year. There is a correlation between the incidence of the disease and age. People younger than 50 years have a low risk of developing herpes zoster which equals approximately 2%. The incidence sharply rises in adults above 50 years; the risk makes up to at least 20% and continues to increase further reaching 35% in people above 80 years [1]. More than 5% of elderly patients have PHN 1 year after acute herpes infection [3]. Herpes zoster is more common for people with immunosuppression caused by HIV, past organ transplantation, cancer, or autoimmune diseases [1]. The predictors of PHN development are as follows: advanced age, acute pain, severe rash, prodromal pain, presence of the virus in peripheral blood, and adverse psychosocial factors [3]. The associated pain syndrome with concomitant allodynia is traditionally attributed to the decrease in the activation threshold of pain-associated neuron clusters[4]. Recently some researchers have shown that TRVP1 receptor activation may also be implicated. This receptor may be a promising target for future analgesic drug development [5].

Antiviral medications (acyclovir, valacyclovir), peroral glucocorticosteroids (GCS), and epidural injections of GCS are used to treat acute herpes zoster. However, treating acute disease does not prevent the development of postherpetic neuralgia [4]. Vaccination for VZV infection, which can increase VZV-specific cellular immunity is a promising strategy for the prevention of herpes zoster and postherpetic neuralgia [1]. First-line therapies for postherpetic neuralgia include antidepressants (amitriptyline, selective serotonin reuptake inhibitors (SSRIs), selective norepinephrine reuptake inhibitors (SNRIs), etc.), anticonvulsants (gabapentin, pregabalin, lamotrigine), and local anesthetics (lidocaine, ropivacaine) applied as creams and patches or used as block injection agents (epidural, regional). Moreover, opioid analgesics (tramadol, morphine, oxycodone), NMDA-agonists (ketamine), high-concentration topical capsaicin, and psychotherapy (cognitive behavioral therapy, biofeedback, and others) are used as adjuvant therapeutic methods [4]. Botulotoxin injections are actively studied as a treatment option for long-lasting PHN [6].

When conservative treatment is ineffective, physicians can try glucocorticoid nerve blocks and pulsed radiofrequency (PRF) of the dorsal root ganglion (DRG). Kotani et al. compared the efficacy of intrathecal administration of methylprednisolone in combination with Marcaine vs. Marcaine injection alone. A 70% reduction in diclofenac use was noted in the group of patients who had been treated with methylprednisolone [7]. There were also attempts at combined epidural administration of methylprednisolone and midazolam in patients with lumbosacral plexus involvement. This led to an increase in the duration of the painless period due to the additional antinociceptive effect of midazolam [7].

The analgesic effect of radiofrequency denervation of dorsal root ganglion on the thoracic level persists for approximately 6 months. Yingwei et al. demonstrated an improvement in patients' quality of life and a reduction in tramadol intake in their study [8]. When these therapies fail to show a significant long-term analgesic effect, the next option is neurostimulation [9].

## 2. Materials and Methods

**Figure 1:**
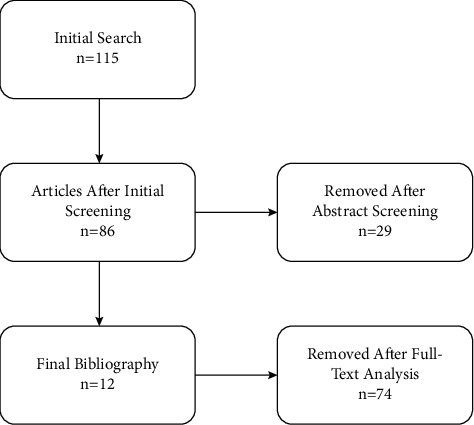
A flowchart describing the search process.

We searched for articles containing the keywords “spinal cord stimulation AND postherpetic neuralgia,” “high-frequency stimulation AND postherpetic neuralgia,” “burst stimulation AND postherpetic neuralgia,” and “dorsal root ganglion stimulation AND postherpetic neuralgia” in Pubmed, Web of Science, and Scopus databases. The search was limited to human studies published in the English language. There were no publication period limitations. Bibliographies and references of selected publications on neurostimulation for PHN were further manually screened. The full text of each article was studied once the abstract was analyzed by the searching reviewer and found appropriate. The initial search yielded 115 articles. Initial screening based on abstract and title allowed us to exclude 29 articles (letters, editorials, and conference abstracts). The full-text analysis allowed us to exclude another 74 articles (fundamental research articles, research utilizing animal subjects, and systemic and nonsystemic reviews) and results of PHN treatment presented with other conditions, leaving 12 articles for the final bibliography (see Figure 1).

Implantation of the system for chronic neurostimulation may be recommended for patients resistant to pharmacological and minimally invasive therapies. There is a series of studies describing the efficacy of peripheral nerve field stimulation (PFNS) and peripheral nerve stimulation (PNS) in regions of pain [10, 11], however, spinal cord stimulation (SCS) is still used more often than other methods in the treatment of PHN with good results [12]. The invention of additional stimulation modes has raised questions about the possibility of improving treatment results, especially in cases where traditional SCS was ineffective or failed to substantially improve quality of life. A variety of stimulation methods are now being tested for the treatment of PHN, with major differences not only in electrode placement but also in core stimulation parameters like pulse frequency. The main types of neurostimulation used for PHN today can be split into the following groups: traditional SCS, high-frequency SCS (HF SCS), burst SCS, and dorsal root ganglion stimulation (DRGS). There is also one report on the combined use of SCS and DRGS [13]. Information regarding the use of alternative stimulation modes is still very limited. The latest (and largest) systematic review by Texicalidis et al. does not discern between various stimulation modes [12]. Reports on the effectiveness of various types of neurostimulation in the treatment of PHN are summarized in Table 1.

## 3. Results

12 articles reporting on the treatment of 134 patients with PHN were analyzed, with a disproportionally large amount of traditional SCS treatment than that to alternative SCS: DRGS (13 patients), burst SCS (1 patient), and high-frequency SCS (2 patients). Long-term pain relief was achieved in 91 patients (67, 9%). The mean VAS score improvement was 61, 4% with a mean follow-up time of 12.85 months. Although the number of patients in alternative SCS studies was very limited, almost all of them showed good responses to therapy with more than 50% VAS improvement and reduction of analgesic dosage. Considering the severity of pain associated with PHN (with an average VAS score of 8 to 9), large randomized-controlled trials comparing alternative and traditional modes of SCS are required, with a substantial amount of PHN patients enrolled.

### 3.1. Traditional Spinal Cord Stimulation

Devices for conventional (tonic) spinal cord stimulation can generate impulses with a frequency range of 2–1200 Hz. The level of electrode implantation is determined by the region of pain, which is usually the same as a dermatome affected by the virus. Pain is also usually accompanied by severe allodynia and discoloration.

There is no need to place an electrode on the level of pain when using HF stimulation or burst stimulation; in these cases, we implant electrodes above the region of pain to overlap pathways of pain signals to higher levels of the nervous system [24].

Contraindications for performing SCS include diffuse pain that is difficult to localize; severe somatic comorbidities; intractable drug addiction, suicidal attempts in the past, and severe mental disorders [24].

### 3.2. SCS Technique

Electrodes are placed under fluoroscopic guidance [2]. Implantation of temporary electrodes is performed before implantation of the neurostimulator to assess the efficacy of SCS in the trial period which usually lasts for 7–10 days. Placement of the whole system is only performed in cases of successful SCS trials.

### 3.3. Tonic Spinal Cord Stimulation

The pulse width of 100–500 *μ*s and pulse frequency of 30–100 Hz is chosen for tonic stimulation. The amplitude is selected for each patient individually based on the comfort level of paresthesias [25]. Due to the specifics of the tonic stimulation mechanism of action, a patient can experience discomfort and even painful paresthesias, which in turn often leads to the inefficiency of SCS in this group of patients with severe allodynia [26]. However, the efficacy of neurostimulation in patients with neuropathic pain associated with acute herpes zoster as well as chronic PHN is confirmed by different studies.

Several studies are describing immediate alleviation of the pain associated with subacute herpes zoster (about 2 months after the onset of the disease) in patients after the SCS trial that has lasted between 7 and 10 days and 2, 5 months. The patients continued to experience pain reduction after 1-year postprocedure. A series of studies demonstrated a complete pain reduction for 1–46 hours during the trial. Some patients with implanted SCS systems had a complete reduction of pain for 2–6 months, others for 3–66 months [9], and in some cases for 50, 8 months [11]. Dong et al. showed the effectiveness of tonic SCS in 32 of 46 patients with postherpetic neuralgia. 18 patients out of 32 had experienced a decrease in pain severity to 2 points assessed by the visual analog scale (VAS). The efficacy of trial stimulation has not differed between patients with different lengths of acute and subacute PHN [2]. Yanamoto and Murakawa also described a significant decrease in pain during the SCS trial. 16 male and 17 female patients were included in the trial (mean age = 73, 1 year). The duration of pain was 2, 6 months. There was a reduction in VAS score from the baseline score of 89, 4 mm to 37, 5 mm in the trial period which lasted for 14, 6 days. As for the results of chronic SCS, the VAS score was 38, 0 mm 3 months postprocedure and 35, 0 mm 6 months postprocedure. 21 of 33 patients (63, 6%) experienced more than a 50% decrease in pain a 1-month postprocedure, 20 of 33 patients (60, 6%) still had this improvement 3 months postprocedure, 21 of 33 (63, 6%) patients–6 months postprocedure. There was no need for a change of therapy or increasing the dosage of analgesic medications in patients with SCS [15].

SCS was also considered a treatment option for acute PHN. Harke et al. described 4 patients who suffered from intolerable acute pain for 1, 8 months. Trial percutaneous electrodes were implanted which lead to immediate pain relief. The baseline VAS score almost immediately reduced from 9, 0 to 1, 0. There was no recurrence of pain syndrome for 13–39 weeks [14]. The interest in nonpharmacological ways of treating pain is especially high in the management of chronic pain in patients with comorbid pathology. The study of treatment of PHN in patients with 3B and IV stages of chronic kidney disease showed the efficacy of SCS. 11 patients with a mean age of 66, 1 ± 4, 5 years and the mean duration of pain equaled 24, 5 ± 5, 2 were included in the study. The level of pain was reduced to 3 points assessed by the VAS. Patients continued to use small doses of medications against neuropathic pain postprocedure [16].

### 3.4. High-Frequency Spinal Cord Stimulation

Conventional spinal cord stimulation generates electrical impulses with a frequency lower than 1200 Hz. The goal of stimulation is to mask patients' feeling of pain by covering the region of pain with paresthesias. Paresthesias are determined as sensations produced by stimulation and perceived as tingling, goosebumps, or vibration. HF10 stimulation uses a frequency of 10000 Hz and differs from tonic SCS by controlling pain without paresthesias [27]. HF10 stimulation is an acknowledged option for the treatment of neuropathic pain syndromes of various etiology [28]. The efficacy of high-frequency stimulation is explained by the desensitization of hyperactive wide dynamic range neurons and control of the «wind-up» phenomenon [29]. No randomized, placebo-controlled trials have been conducted to investigate the effectiveness of high-frequency neurostimulation in the treatment of postherpetic neuralgia. Shechter et al. published the results of the comparative study of high-frequency SCS vs. low-frequency SCS in the rat model. The researchers concluded that high frequencies (1000 Hz and above) of spinal cord stimulation provided earlier suppression of allodynia than conventional SCS with 50 Hz frequency [30]. There are several cases describing the effectiveness of high-frequency neurostimulation in postherpetic neuralgia. Sai et al. presented the case of a 70-year-old man who had undergone implantation of a spinal cord stimulation system to treat postherpetic thoracic neuralgia (Th5–Th10). The baseline level of pain intensity was 10 out of 10 points on VAS. After implantation, the parameters were set as follows: frequency-60 Hz, pulse width-250–400 microseconds, amplitude-0–3 mA. An additional program with a frequency of 10000 Hz was also installed. The patient preferred the latter over other programs. 7 days after the surgery, the patient remarked an 85–90% reduction in pain at high frequencies, along with a significant reduction in the frequency and intensity of pain attacks and improvement of sleep [20]. Tang et al. reported a case of a 56-year-old man with severe pain in the right upper limb area. The electrode was placed in the dorsal root entry zone. The authors tried different parameters of SCS, initially, the high frequency of 360 Hz was chosen, but then the stimulator was programmed at 40 Hz. Within 7 days there was a remarkable drop in neuropathic pain from 9 points to 2 points on VAS [21]. Lerman et al. also presented a case of subcutaneous implantation of the peripheral nerve stimulator that resulted in an 80% pain regression with high-frequency programs of 1200 Hz and 100 Hz in a 52-year-old man with drug-resistant postherpetic neuralgia in the area of the first branch of the trigeminal nerve [31].

### 3.5. Burst Spinal Cord Stimulation

Burst stimulation is a type of stimulation in which impulses are delivered in series of 5, with each impulse lasting 100 *μ*sec at 500 Hz frequency with a total duration of each burst of 1 msec. The frequency of the bursts is 40 Hz. Passive repolarization happens between the bursts and lasts for about 5 msec. [24, 25, 32]. Passive repolarization occurs before the next burst and lasts for 5 seconds. This pulse-free period is called an interburst interval. The amplitude is chosen to be at the level of subthreshold stimulation providing pain relief without paresthesias [24, 25]. Burst and tonic stimulation differ in types of cellular response. Burst stimulation in contrast to tonic stimulation affects non-GABA receptors and acts on the level of dorsal horns of the spinal cord. It also does not activate paresthetic pathways, while tonic stimulus enables pain impulses to enter the thalamus which causes paresthesias. A probable additional mechanism of analgesic effect in burst stimulation is the activation of anti-inflammatory cytokine interleukins (IL-10), which increase was noted in the cerebrospinal fluid and blood [33–35]. Besides the analgesic effect, IL-10 enhances nerve fibers regeneration [36]. We found a single case of an 80-year-old man with postherpetic neuralgia presented with severe pain (VAS score = 8) in the area of the second left thoracic dermatome. After the implantation of the leads for the spinal cord stimulation on the Th1–Th4 level, the patient experienced significant alleviation of pain. The VAS score was reduced to 3 points and the patient preferred burst mode over tonic mode during the trial. 6 months after the implantation of the permanent stimulator the patient reported 50% pain relief [22].

### 3.6. Dorsal Root Ganglion Stimulation (DRGS)

Studies show that DRGS plays a key role in the management of nociceptive as well as neuropathic pain. The method is especially useful for the treatment of the pain in cases when conventional spinal cord stimulation is not effective enough, primarily due to the difficulties in getting paresthesias in strictly limited areas such as the groin area, the outer side of the foot, or specific intercostal space [24]. One of the main analgesic mechanisms of DRGS is the selective activation of *Aβ-*, *Aδ*-, and C-fibers when applying low frequencies. Generating action potential at very low frequencies, DRGS enables the blocking of pain stimuli through the activation of opioid receptors without engaging of GABA system [37]. Moreover, hyperpolarization of C-fibers membranes happens through calcium-activated potassium channels in T-shaped branches of primary sensory neurons which prevents pain stimuli from reaching the central nervous system [38]. Kim et al. published the results of the study of 69 patients with medically resistant postherpetic neuralgia who have undergone the implantation of the DRGS system. The mean interval between the onset of herpes zoster and the surgery was 21, 9 months. DRGS was performed at cervical, dorsal, and lumbar levels. Pain intensity assessed by VAS reduced from 6, 9 to 2, 7 points in 3, 5 months. The decrease of morphine equivalent daily dose from 35, 2 mg to 20 mg after the surgery was noted in one case [39]. This study also showed that 3 of 49 (6.1%) patients stopped taking analgesic medications, while all others the patients significantly decreased their drug intake [12, 40].

A clinical case of an 80-year-old man with 15 years duration of pain in the left side of the head and neck in the area of the C2 dermatome was described by Lynch et al. The patient has permanent severe pain with no response to multimodal therapy including tramadol 50 mg 2 times per day, pregabalin 75 mg 3 times per day, hydrocodone-acetaminophen 5/500 3 days per day, and facet joint block. The patient was referred to implantation of the lead for the test stimulation. After the surgery, the level of pain decreased by 50% and the patient stopped taking analgesic drugs. A reduction of 20% was noted in 6 months postoperatively and the patient still did not need any medications [41].

Hunter et al. in the study of DRGS for various etiologies described 4 patients with PHN whose mean reduction of pain was 81, 2% assessed by NRS and a successful trial rate was 75% [17]. However, some unsatisfying results of DRGS for the management of PHN were also reported. Anthony et al. described 3 patients with PHN who underwent thoracic DRGS with no or minimal pain improvement postoperatively [18]. Piedade et al. presented another two cases of PHN patients who did not respond well enough to cervical and high thoracic DRGS [19].

## 4. Discussion

Herpes zoster and postherpetic neuralgia associated with it are common diseases that severely impact the quality of life and cause the progression of disability in elderly people. Treatment of PHN is usually a challenge since it is often unresponsive to traditional management options. Vaccination against herpes zoster infection can decrease the risks of having an infection and the severity of its clinical presentation. Spinal cord stimulation has been used for over 50 years for the treatment of pain syndrome. The effective analgesic effect of SCS has been proven by numerous studies. Neurostimulation is especially relevant for patients with severe comorbidities. Considering high efficacy, minimal invasiveness of neurostimulation, and ability to control analgesic effect postprocedure, it can be applied as one of the first steps in the management of neurogenic pain syndromes. However, physicians should strictly follow inclusion criteria, indications, and contraindications for the treatment to avoid negative results in the postoperative period. Novel stimulation strategies such as burst stimulation and high-frequency stimulation could be a possible solution to the treatment of severe cases of PHN due to their ability to provide pain relief without unpleasant paresthesias. Lately, dorsal root ganglion stimulation becomes a popular method of PHN management. DRGS of specific ganglions does not cause paresthesias in the regions that are not affected by pain, which is especially important for patients with allodynia as a symptom of PHN. Despite some promising results demonstrating a significant decrease in pain intensity after DRGS, there were also conflicting reports on its efficacy. Controlled trials with larger cohorts are needed to conclude the efficiency of different types of stimulation.

## Figures and Tables

**Table 1:** Literature review summary.

Reference number	Year of publication	Comments	Type of stimulation	Number of patients	Number of patients with long-term pain relief
[2]	2017		stSCS	46	32
[14]	2002		SCS	28	23
[15]	2012		stSCS	33	21
[16]	2011		SCS	11	4
[17]	2019	DRG focus trial	DRGS	4	3
[18]	2019		DRGS	3	0
[19]	2029		DRGS	2	1
[20]	2021		hfSCS	1	1
[21]	2018		hfSCS	1	1
[22]	2021		burstSCS	1	1
[23]	2021		DRGS	3	3
[13]	2021		SCS + DRGS	1	1
% Of patients with long-term pain relief	Affected dermatomes	Average pain reduction (%)	Follow-up, mo	Medication usage after stimulation	
69.6	T5-L5	50.7	6–12	Reduction or discontinuation in 11 patients	
82.1	C3-S1	89	29 average	Discontinuation in 13 patients	
63.6	Cervical and thoracic	49	6	No patient required changes in drug doses	
36.3	C5-L2	N/A	34.5	N/A	
75	N/A	82.5	N/A	N/A	
0	T1-L2	16,00	8	No changes	
50	C8-T7	37.5	12	N/A	
100	T2	90	7 days	N/A	
100	C4-T1	78	N/A	Reduction of oxycodone usage	
100	T2	62.5	6	Reduction in opioid dose	
100	T5-T10	60	18	Reduction or discontinuation in all patients	
100	C6-T1	60	2	Discontinuation of fentanyl usage	

## Data Availability

No data were available for this study.
